# Starvation-Induced Changes to the Midgut Proteome and Neuropeptides in *Manduca sexta*

**DOI:** 10.3390/insects15050325

**Published:** 2024-05-02

**Authors:** Gurlaz Kaur, David R. Quilici, Rebekah J. Woolsey, Juli Petereit, Andrew B. Nuss

**Affiliations:** 1 Cell and Molecular Biology Graduate Program, University of Nevada, Reno, NV 89557, USA; gurlaz@ualberta.ca; 2 Mick Hitchcock, Ph.D. Nevada Proteomics Center, University of Nevada, Reno, NV 89557, USA; quilici@unr.edu (D.R.Q.); rebekahw@unr.edu (R.J.W.); 3 Nevada Bioinformatics Center, University of Nevada, Reno, NV 89557, USA; jpetereit@unr.edu; 4 Department of Agriculture, Veterinary & Rangeland Sciences, University of Nevada, Reno, NV 89557, USA

**Keywords:** feeding, endocrine, peptidome, tobacco hornworm, lepidoptera

## Abstract

**Simple Summary:**

An optimal internal response to feeding state is critical for survival and development in insects. Part of this response originates in the insect midgut, which detects the nutritional status of incoming food and sends relevant signals to other organs via hormones. Starvation leads to changes in insect behaviors, such as food-seeking, and physiology, such as modifying nutrient usage to save energy for survival. Therefore, in this study, proteomics was used on fed and starved tobacco hornworm (*Manduca sexta*) caterpillar digestive tracts to characterize how the midgut responds to starvation. Several processes were modified by starvation, including digestive enzymes and metabolic functions. Additionally, gut-produced neuropeptide hormones were detected, and some changed in abundance in starved insects, particularly neuropeptide F1, which suggests that the dynamics of neuropeptide F1 are tied to feeding state. Overall, this study provides a foundation for understanding the gut response, particularly the dynamics of neuropeptide hormones, to starvation and provides multiple targets for future work.

**Abstract:**

Starvation is a complex physiological state that induces changes in protein expression to ensure survival. The insect midgut is sensitive to changes in dietary content as it is at the forefront of communicating information about incoming nutrients to the body via hormones. Therefore, a DIA proteomics approach was used to examine starvation physiology and, specifically, the role of midgut neuropeptide hormones in a representative lepidopteran, *Manduca sexta*. Proteomes were generated from midguts of *M. sexta* fourth-instar caterpillars, starved for 24 h and 48 h, and compared to fed controls. A total of 3047 proteins were identified, and 854 of these were significantly different in abundance. KEGG analysis revealed that metabolism pathways were less abundant in starved caterpillars, but oxidative phosphorylation proteins were more abundant. In addition, six neuropeptides or related signaling cascade proteins were detected. Particularly, neuropeptide F1 (NPF1) was significantly higher in abundance in starved larvae. A change in juvenile hormone-degrading enzymes was also detected during starvation. Overall, our results provide an exploration of the midgut response to starvation in *M. sexta* and validate DIA proteomics as a useful tool for quantifying insect midgut neuropeptide hormones.

## 1. Introduction

The insect midgut is a complex organ specialized not only for digestion and nutrient absorption but also as a sensory surface to detect nutrients in the gut lumen [1]. The midgut, as an endocrine organ, can communicate this information with local digestive cell populations and with other organs via enteroendocrine cells that release hormones into the hemolymph [2,3]. Many of these hormones are neuropeptides and may coordinate digestion, send feedback to the central nervous system (CNS), and regulate metabolism based on gut stimuli [3]. Conversely, a lack of nutrients in the midgut, starvation, elicits a signaling response from the midgut, or a halt to ongoing neuropeptide hormone release, that may induce responses to conserve energy or urgently seek food sources [4].

The expansion of omics techniques has greatly aided in identifying neuropeptides from various insect species in recent decades. The number of neuropeptides encoded in insect genomes varies greatly, but most have a core set of approximately 50 neuropeptide families represented [5,6]. Insect midgut endocrine cells produce at least 10–11 of these peptide hormones including allatostatins, bursicon, CCHamides, diuretic hormones, insulin-like peptides, tachykinin, short neuropeptide F, neuropeptide F (NPF), and orcokinin, depending on the species [2,3,7,8]. The production and release of neuropeptide hormones is responsive to gut contents, starvation, and levels of circulating macronutrients, and these neuropeptide hormones have downstream physiological and behavioral impacts [3,9,10,11,12]. For instance, courtship is suppressed, and food-seeking is heightened in starved male *Drosophila melanogaster* via the following progression: Consumption of amino acids induces release of diuretic hormone 31 (DH31) from enteroendocrine cells. This circulating, midgut-derived DH31 stimulates DH31 receptor-expressing cells of the brain, including allatostatin-C-producing cells that inhibit feeding and corazonin-expressing cells that restore courtship behavior [11].

The tobacco hornworm, *Manduca sexta*, has long served as a model for lepidopteran physiology and has facilitated a fundamental understanding of insect immunity, apoptosis, metamorphosis, and serine proteases, among other functions [13,14]. Starvation-induced effects on survival and physiology have also been previously explored in *M. sexta*. In the final (fifth) instar, *M. sexta* larvae typically take 8 days to develop until pupation with optimal nutrition [15], with feeding ceasing on day 5 at the onset of the wandering stage [16]. However, prior to the wandering stage, they can survive for up to 5–6 days in the fifth instar without food, with sufficient hydration [15]. Initially, *M. sexta* larvae retain glycogen reserves in the fat body until 24 h of starvation and then gradually start using these reserves for gluconeogenesis [17]. As starvation progresses through 48 h, the pentose phosphate pathway enhances glucose and fructose-6-phosphate [18]. Also, after 48 h of starvation, lipid synthesis in *M. sexta* ceases, and the pyruvate carboxylase arm of the TCA cycle is enriched over pyruvate dehydrogenase [18]. This switch in the TCA cycle under starvation conditions leads to increased glucose synthesis over lipids to maintain trehalose levels in the hemolymph. However, trehalose levels usually drop in starved caterpillars after 72 h and are barely detectable [15]. These physiological changes are mediated in part by circulating nutrients but also by signaling molecules between organs [19]. However, much remains to be explored regarding the coordination of these physiological changes and the role of tissues such as the midgut in the starvation response.

The lepidopteran midgut plays an important and complex role in nutrient metabolism, yet changes to the physiology of this organ during starvation are relatively unexplored on a proteomic level. Therefore, a label-free data-independent acquisition (DIA) proteomics approach was used on midguts of 24 h and 48 h starved fourth-instar *M. sexta* larvae compared to corresponding fed controls. A total of 3047 proteins were identified and a total of 854 of these were significantly different in abundance in different starvation timepoint comparisons. In total, 132 proteins were consistently different in abundance between fed and starved midguts over all timepoints, and KEGG analysis was used to determine overarching pathways that were more, or less, abundant. In general, starvation induced a shift from digestive enzymes, mitochondrial and ribosomal activity, and metabolism to oxidative phosphorylation, mitophagy, and nucleotide-processing proteins. In addition, six neuropeptide hormones or related signaling cascade molecules were detected, of which NPF1 was the most abundant, and the abundance of several of these neuropeptides significantly increased under starvation conditions. These results add to our understanding of larval midgut physiology and, especially, the effects of feeding state at the protein level. The differently abundant proteins, especially the neuropeptides, can be further explored for their independent roles in insect physiology in future studies.

## 2. Materials and Methods

### 2.1. Identification of Neuropeptide Genes

A list of neuropeptides for *M. sexta* was assembled using search inputs reported from other insects including *Chilo suppressalis* [20], *Bombyx mori* [21], *Spodoptera exigua* [22], and the Database for Insect Neuropeptide Research (DINeR) [5] (Table S1). Neuropeptide names were used to search annotated orthologs from the *M. sexta* reference genome, JHU_Msex_v1.0, on the NCBI database [23]. For neuropeptides that were not detected by the above methods, BLASTn and tBLASTn searches were performed by using the NCBI BLAST web interface [24] on the *M. sexta* reference genome using neuropeptide sequences from other lepidopterans.

### 2.2. Sample Collection

*Manduca sexta* eggs were obtained from Great Lakes Hornworm (Romeo, MI, USA), and caterpillars were reared in individual containers on a wheat germ-based artificial diet (Table S2) (derived from [25,26,27,28,29,30]) at 28 °C and 40% relative humidity with a 16:8 h light:dark photoperiod. Third-instar caterpillars (head capsule width 3.1 to 3.5 mm [31]) were monitored at the head slip transition stage for molting. Newly eclosed 4th-instar caterpillars were fed for 24 h and then split into “starved” or “fed” 24 h and 48 h groups. Caterpillars were housed in individual rearing cups with a wide screen mesh base to allow frass to drop from the cage, preventing starved caterpillars from feeding on the frass. 1% agarose blocks were provided to starved caterpillars to prevent dehydration, and fed caterpillars were provided with fresh artificial diet. Midguts from respective fed and starved caterpillars were dissected in PBS at 24 h and 48 h. Dissected midguts were ground in 50% acetonitrile and lyophilized in a freeze dryer (HarvestRight, Salt Lake City, UT, USA) in 1.7 mL centrifuge tubes with holes poked in the lids. The lyophilized protein vials were sealed with parafilm (Thermo Scientific, San Jose, CA, USA) and stored at −80 °C.

Freeze-dried midgut samples were resuspended in Thermo Scientific EasyPep Mini MS Sample prep kit (Cat #A40006) lysis buffer, and protein content was estimated using the fluorescence-based protein assay EZQ (Invitrogen #R33200). Then, 100 µg protein extracts were reduced, alkylated with iodoacetamide, and digested with a trypsin/Lys-C protease mixture using a Thermo Scientific EasyPep Mini MS Sample prep kit (Cat #A40006) by following the manufacturer’s protocol. The trypsin/Lys-C was added 1:10 (enzyme:protein) for digestion. Samples were purified for analysis using the column provided with the kit. Samples were reconstituted in 100 µL of 0.1% formic acid in water, with a final concentration of 1 µg/µL for analysis.

### 2.3. Liquid Chromatography

Samples were analyzed using an UltiMate 3000 RSLCnano system (Thermo Scientific, San Jose, CA, USA). The peptides were trapped prior to separation on a 300 µm i.d. × 5 mm C18 PepMap 100 trap (Thermo Scientific, San Jose, CA, USA) for 5 min at 10 µL/min. Separation was performed on a 50 cm uPAC C18 nano-LC column (PharmaFluidics, Ghent, Belgium) on an EasySpray source (Thermo Scientific, San Jose, CA, USA) fitted with a 30 µm ID stainless steel emitter (PepSep, Marslev, Denmark). Separation was performed at 350 nL/min using a gradient from 1 to 45% for 60 min (Solvent A: 0.1% formic acid; Solvent B: acetonitrile, 0.1% formic acid). 

### 2.4. Mass Spectrometry

Data Independent Analysis (DIA) was performed by using an Eclipse Tribrid Orbitrap mass spectrometer (Thermo Scientific, San Jose, CA, USA) [32,33]. Aliquots from each of the biological samples were combined to form a biologically pooled sample. This pooled sample was then used to generate a hybrid chromatographic library consisting of DIA and DDA spectra using the Spectronaut-integrated database search engine Pulsar. This hybrid library was generated using a combination of six DIA gas phase fractions (GPF) and three full-scan DDA spectra of the biological sample pool. The DIA GPF’s used 4 *m*/*z* precursor isolation windows in a staggered pattern (GPF1 398.4–502.5 *m*/*z*, GPF2 498.5–602.5 *m*/*z*, GPF3 598.5–702.6 *m*/*z*, GPF4 698.6–802.6 *m*/*z*, GPF5 798.6–902.7 *m*/*z*, and GPF6 898.7–1002.7 *m*/*z*) at a resolution of 60,000. The AGC target was set to custom with a normalized target of 1000%; a maximum injection time was set to dynamic with a minimum of nine points across the peak; and an NCE of 33 using higher-energy collision dissociation (HCD). The three DDA full scan runs were performed with an MS1 precursor selection range from 375 to 1500 *m*/*z* at a resolution of 120 K with a normalized automatic gain control (AGC) target of 250% and an automatic maximum injection time. Quadrupole isolation of 0.7 Th was used for MS2isolation and CID fragmentation in the linear ion trap with a collision energy of 35% and a 10 ms activation time. The MS2 AGC was in standard mode with a 35 ms maximum injection time. The instrument was operated in a data-dependent mode with a 3 s cycle time and the most intense precursor priority, and the dynamic exclusion was set to an exclusion duration of 60 s with a 10 ppm tolerance. Biological samples were run on an identical gradient to that of the GPFs using a staggered window scheme of 8 *m*/*z* over a mass range of 385–1015 *m*/*z*. Precursor isolation was performed in the Orbitrap at a 60,000 resolution with a dynamic maximum injection allowing for a minimum of nine points across the peak, a custom AGC normalized to 1000%, and an NCE of 33 using HCD. The species-specific FASTA database for *M. sexta* (RefSeq), containing 25,012 proteins, and a database of known contaminants (Crap_uniprot_with_human_MRSonbeadV2) were downloaded from RefSeq and Uniprot, respectively. The variable modifications considered were as follows: Carbamidomethylation C. Identification cutoffs for precursor and protein Q-value cutoffs were set to 0.01, and quantity was based on the area of MS2 ions. A global imputing strategy was used for missing values.

### 2.5. Data Analysis

An initial data analysis was performed using the Spectronaut™ software (version 15.7.220308.50606 Rubin, Biognosys AG, Schlieren, Switzerland). Raw data from the Spectronaut software were exported as raw intensities to R and analyzed for differential protein abundances. Total protein intensities across biological replicates within all treatments were similar. Raw intensities were transformed and compared using five normalization methods, and cyclic loess log2-normalized data provided the most consistent results and were used to calculate protein abundances and differential analysis. Principal component analysis (PCA) was plotted for log2-transformed protein abundances to compare the distribution of different replicates and to gauge treatment group similarity. Fed and starved samples segregated into distinct groups. However, separation by time within starved or fed groups was less distinct: starved 24 h and 48 h protein samples were tightly clustered, while fed 24 h and 48 h samples had a wide, overlapping spread that did not segregate into discrete 24 h and 48 h clusters (Figure S1). Reproducibility within biological groups was also evaluated by Pearson correlation coefficient analysis, and the samples collected at the same timepoint within the fed and starved treatments (r > 0.95, Pearson) were highly correlated with a few exceptions (starved 24 h replicate 3, and fed 24 h replicate 3).

Therefore, a differential analysis to compare fed and starved midgut samples was performed at three levels: 24 h, 48 h, and combined 24 h and 48 h, by using Limma [34]. These analyses provided an overall fed vs. starved (combined) and individual timepoint (24 h and 48 h) quantitative proteomic profile.

Protein IDs that were differentially abundant in fed and starved conditions were used to identify Gene IDs from the *M. sexta* reference genome [23]. KEGG pathways for *M. sexta*, along with their associated gene ids, were imported to R by using KEGGREST [35]. A KEGG Gene Set Enrichment Analysis (GSEA) was performed using clusterProfiler (v4.2.2) [36] with a minimum gene set size of 3 and a *p*-value cutoff of 1. Gene set enrichment figures were plotted using ggplot2 (RStudio 2021.09.2+382 “Ghost Orchid”). Venn diagrams were plotted by using Venny2.1 [37].

## 3. Results

### 3.1. Differential Proteomics

**Figure 1:**
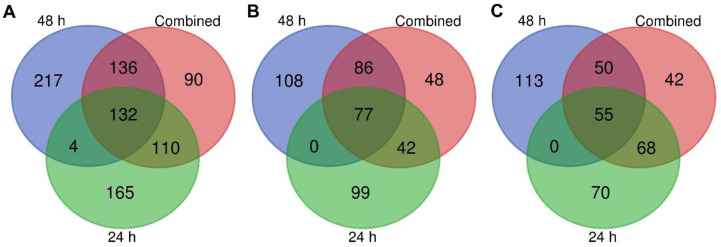
Venn diagrams illustrating differentially abundant proteins between combined 24 h and 48 h fed vs. starved (red), 24 h fed vs. 24 h starved (green), and 48 h fed vs. 48 h starved (blue) analyses, significant at FDR< 0.05. (**A**) In total, 854 proteins had a significant change in abundance between the three analyses, (**B**) subset of proteins (460) with higher abundance in fed samples, (**C**) subset of proteins (398) with higher abundance in starved samples.

**Figure 2:**
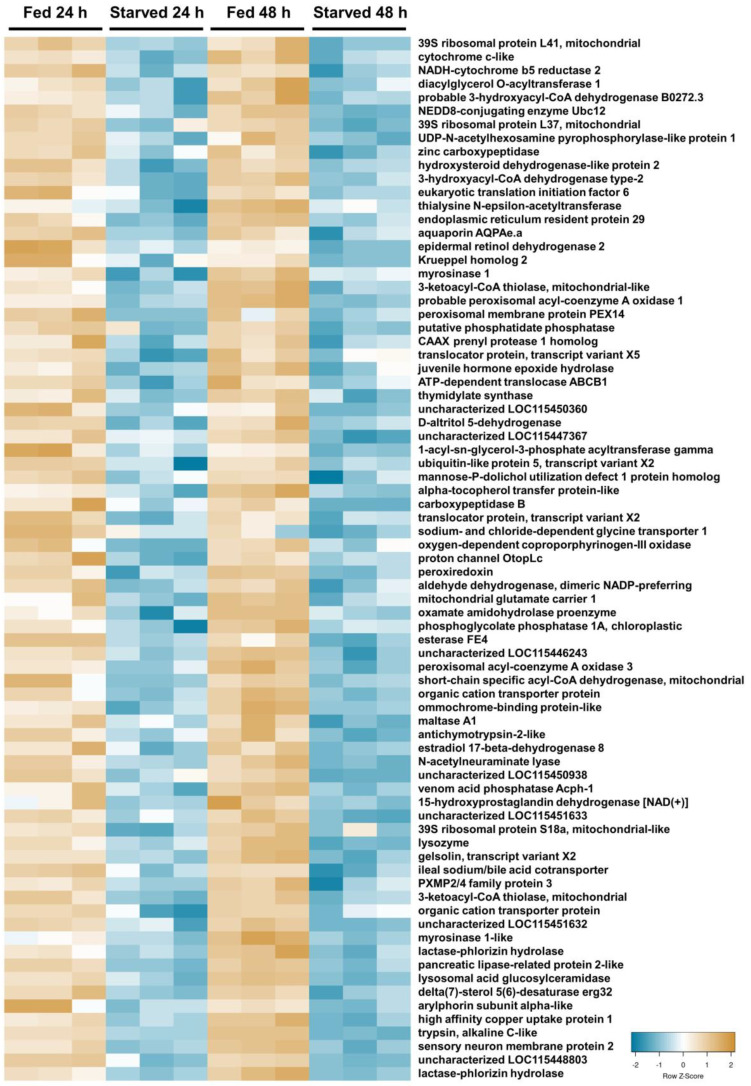
Heatmap illustrating 77 midgut proteins with significantly lower abundance in samples of fourth-instar *M. sexta* caterpillars starved for 24 h and 48 h versus caterpillars fed for 24 h and 48 h. Only common significant proteins (adjusted *p*-value ≤ 0.05) across all three comparisons are depicted here. Protein abundance was scaled to Z-scores for plotting. Blue color indicates a lower abundance in starved caterpillars, and tan color indicates a higher abundance.

**Figure 3:**
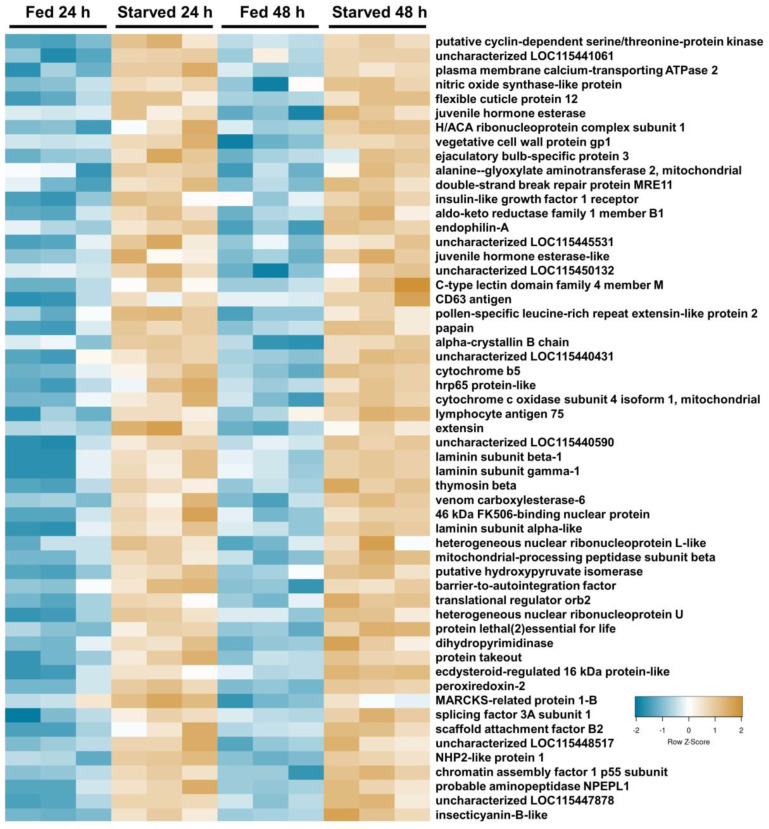
Heatmap illustrating 55 midgut proteins with significantly higher abundance in samples of fourth-instar *M. sexta* caterpillars starved for 24 h and 48 h versus caterpillars fed for 24 h and 48 h. Only common significant proteins (adjusted *p*-value ≤ 0.05) across all three comparisons are depicted here. Protein abundance was scaled to Z-scores for plotting. Blue color indicates a lower abundance in starved caterpillars, and tan color indicates a higher abundance.

To characterize the starvation response in the midgut proteome of fourth-instar *M. sexta*, 24 h and 48 h starved larval midguts were compared with fed 24 h and 48 h controls using DIA mass spectrometry. A total of 3047 proteins were identified from the 12 midgut protein samples. Based on the PCA analysis, the clustering of samples was discrete by feeding status but not discrete by time (Figure S1), and therefore three comparisons were made: (1) 24 h fed vs. 24 h starved; (2) 48 h fed vs. 48 h starved; and (3) combined 24 h and 48 h fed vs. combined 24 h starved and 48 h starved. A total of 854 unique proteins displayed a significant change in abundance in one or more of the analyses (Figure 1). However, only 132 proteins consistently differed in abundance across all three analyses of starved midguts compared with the fed controls (Figure 1A). Out of these, 77 proteins were more abundant in the fed samples (Figure 1B and Figure 2), while 55 were more abundant in starved samples (Figure 1C and Figure 3; Table 1). An additional 722 proteins were identified as significantly different in abundance in one or two of the analyses (Figure 1A; Table S3).

Of the 132 differentially abundant proteins, several annotations suggested an association with digestion, metabolism, or mitochondrial function (Table 1; Figure 2 and Figure 3). Seven proteins were recognized as being associated with protein degradation (trypsins, proteases, and peptidases), three facilitate sugar digestion (two lactases and one maltase), and two were associated with lipid digestion (lipase and carboxylesterase). These were predominantly more abundant in fed caterpillar midguts (Table 1). Another 10 proteins associated with glycerol or assorted dehydrogenases (likely involved in metabolism) were all more abundant in fed caterpillars. Nine proteins were identified as mitochondrial proteins and were also predominantly more abundant in fed caterpillars. In addition to the above, 42 protein digestion enzymes (15 up and 28 down (trypsin alkaline A both up at 24 h and down at 48 h)), 6 sugar digestion enzymes (all down), and 10 lipid digestion enzymes (two up and eight down) were significantly different between fed and starved midguts in one or two analyses. An additional 30 metabolic enzymes (5 up and 25 down) and 74 mitochondrial proteins (26 up and 48 down) were also significantly different between fed and starved midguts in one or two analyses.

In addition to the above-mentioned proteins, a significant change in abundance was notable in a few other groups of proteins. For instance, three juvenile hormone (JH)-degrading enzymes significantly changed in abundance between feeding states in all three analyses (Table S3), and an additional five (three up and two down) in one or two analyses. Also, three cytochromes (one more abundant and two less) changed in abundance under starvation in all analyses. An additional three cytochromes were significantly less abundant in one or two analyses. Finally, alpha, beta, and gamma subunits of laminin, a component of the midgut basement membrane, were all significantly more abundant in starved caterpillars in all analyses. Additionally, eight laminin-like proteins, nidogen, collagen, collagenase, and integrin proteins were significantly different in abundance in one or two analyses (four up and four down).

### 3.2. KEGG Enrichment Analysis

**Figure 4:**
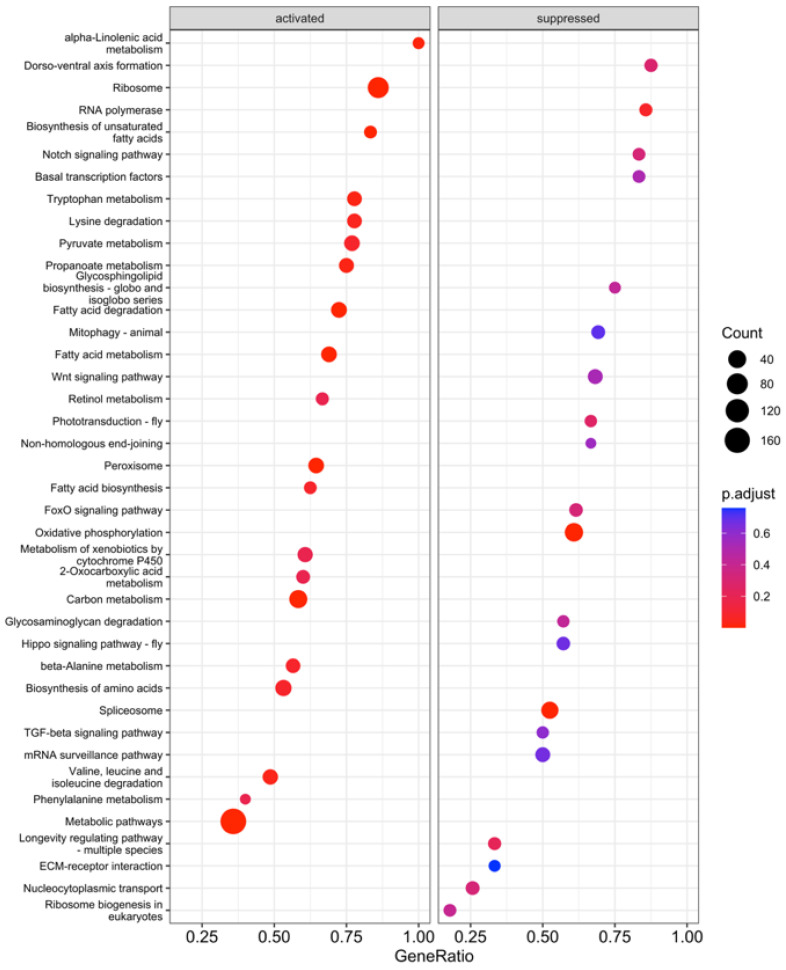
KEGG pathway Gene Set Enrichment Analysis (GSEA) of *M. sexta* midgut proteomic data between fed and starved fourth-instar caterpillars in the combined 24–48 h analysis. “Activated” pathways were significantly higher in abundance in fed samples, and “suppressed” pathways were more abundant in starved midguts.

**Figure 5:**
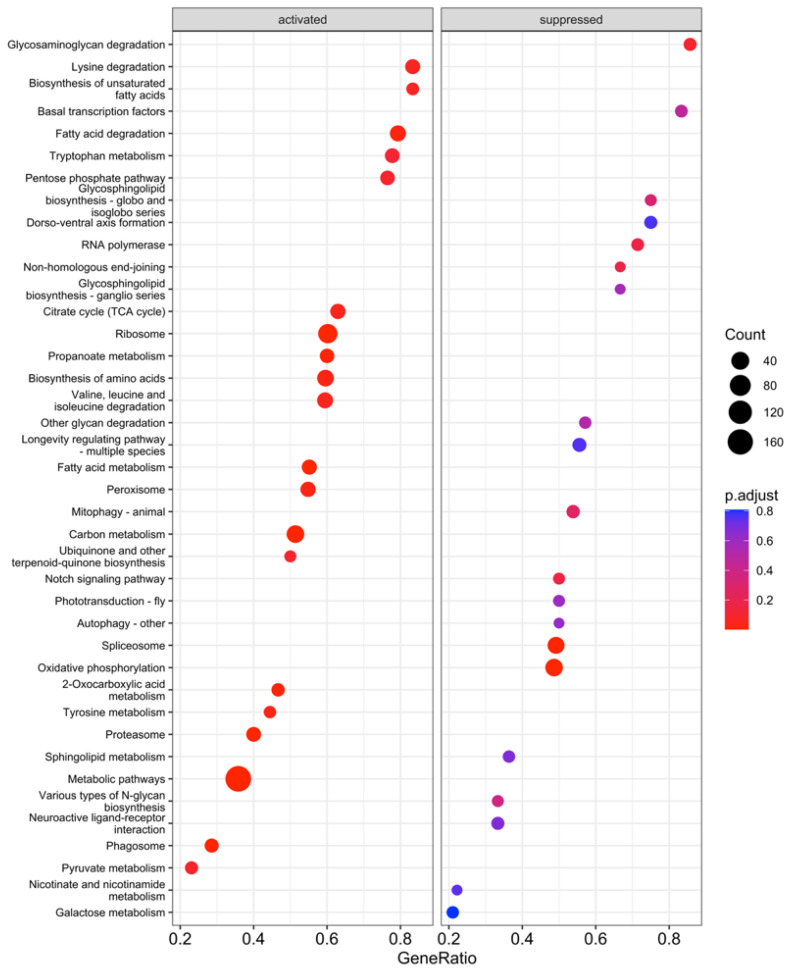
KEGG pathway Gene Set Enrichment Analysis (GSEA) of *M. sexta* midgut proteomic data between fed and starved fourth-instar caterpillars in the 24 h analysis. “Activated” pathways were significantly higher in abundance in fed samples, and “suppressed” pathways were more abundant in starved midguts.

**Figure 6:**
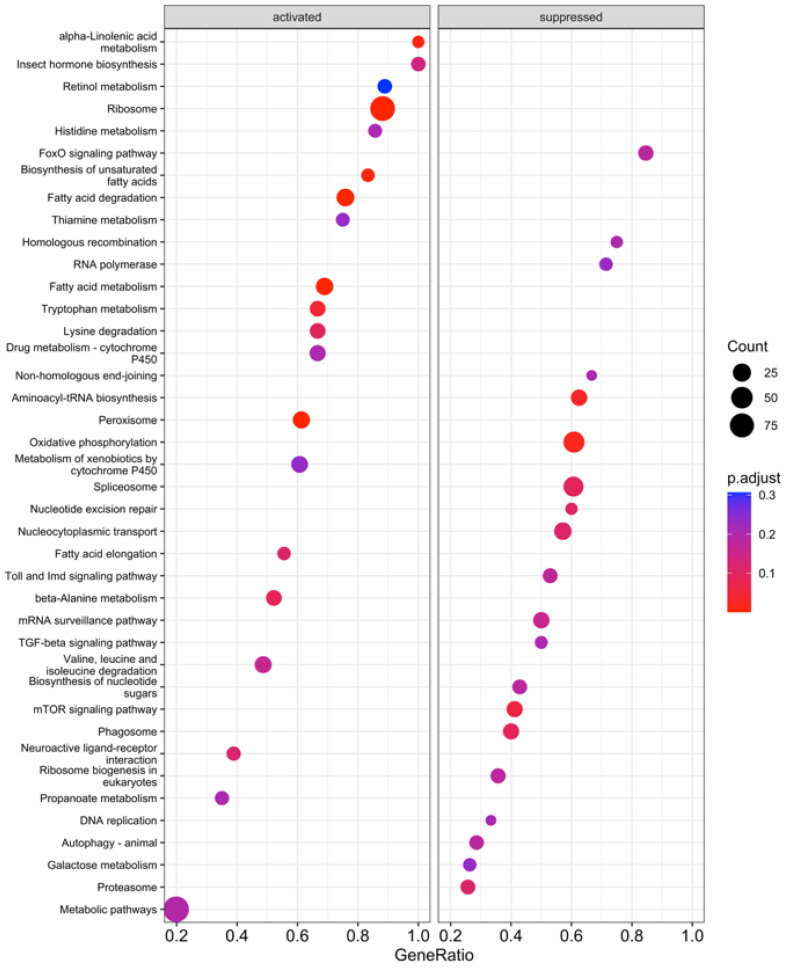
KEGG pathway Gene Set Enrichment Analysis (GSEA) of *M. sexta* midgut proteomic data between fed and starved fourth-instar caterpillars in the 48 h analysis. Activated pathways were significantly higher in abundance in fed samples, and suppressed pathways were more abundant in starved midguts.

KEGG enrichment analysis was performed on the proteome samples to provide insight into larger-scale processes in the midgut impacted by starvation. As with the DIA analysis, three comparisons were made: (1) 24 h fed vs. 24 h starved; (2) 48 h fed vs. 48 h starved; and (3) combined 24 h and 48 h fed vs. combined 24 h starved and 48 h starved. A total of 67 KEGG pathways were significantly different between the fed and starved samples across all comparisons, and 14 of these were consistent across all three comparisons (Figure 4, Figure 5 and Figure 6). Of these 14, 10 were suppressed during starvation, while 4 were activated. The largest impacts were on metabolism: two broad categories, metabolic pathways and fatty acid metabolism, were less abundant in starved midguts, along with the more specific categories of valine, leucine and isoleucine degradation; lysine degradation; tryptophan metabolism; fatty acid degradation; biosynthesis of unsaturated fatty acids; propanoate metabolism; and proteins of the peroxisome. Conversely, oxidative phosphorylation pathway proteins were more abundant in starved caterpillars. An additional 33 pathways involving metabolism, degradation, or biosynthesis were significantly different in one or two of the analyses. The mitophagy pathway was also significantly more abundant in starved caterpillars in the combined and 24 h comparisons.

In addition to impacts on metabolism, starved caterpillars also had significantly more proteins for three nucleotide-processing KEGG pathways, including those involved with RNA polymerase, the spliceosome, and non-homologous end-joining pathways, across all three analyses. Pathways involved with the ribosome, however, were significantly less abundant across all three analyses. An additional five KEGG pathways involved with nucleotide processing had significantly more abundance in one or two of the analyses. Also, signaling pathways were identified as significantly more abundant in 10 KEGG pathways in starved midguts. These were significant for one or two of the analyses but never for all three.

### 3.3. Neuropeptide Hormones in the Midgut Proteome

Changes in neuropeptide hormone abundance in response to starvation were specifically scrutinized in this study. To examine this, a list of 69 neuropeptides, as annotated in the *M. sexta* reference genome (JHU_Msex1.0), was assembled (Table S1). Most insect neuropeptide families were represented in the *M. sexta* genome, with the exceptions of arginine–vasopressin-like peptide, neuropeptide-like precursor 2 (NPLP2), proctolin, glycoprotein hormones, leucokinin, neuroparsin, pigment dispersing factor (PDF), and sulfakinin. Transcriptomes from larval midguts were data mined [14] and neuropeptide hormone expression was estimated based on FPKM values (Table 2). Not all gene IDs from this annotation have survived the more recent annotation of the *M. sexta* reference genome (JHU_Msex1.0). To confirm whether neuropeptides from the most current *M. sexta* genome annotation were truly missing or just unannotated, BLASTn and tBLASTn on the NCBI web interface was used to probe the JHU_Msex1.0 genome assembly using queries based on previous *M. sexta* neuropeptide annotations or based on *Chilo suppressalis* [20], *Bombyx mori* [21], or *Spodoptera exigua* [22] neuropeptides.

**Figure 7:**
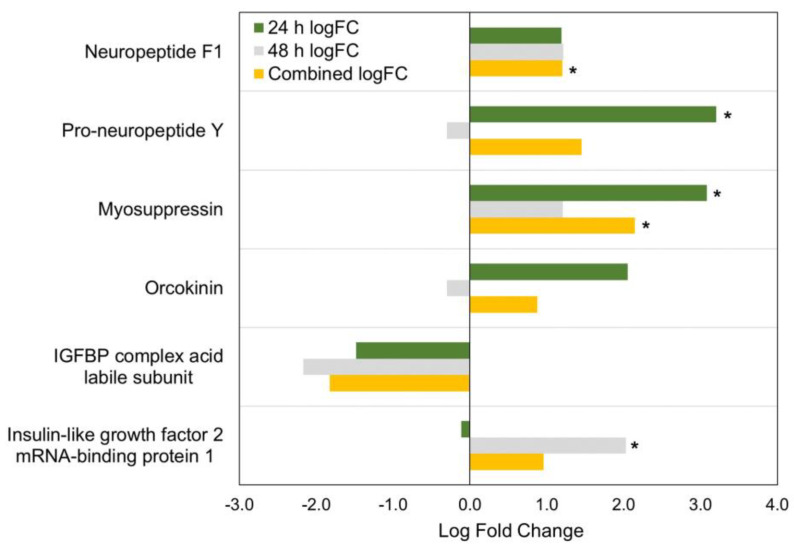
Log fold changes (logFC) in peptide hormones and neuropeptide-interacting proteins detected in *M. sexta* midgut samples. Positive logFC represents higher abundance in starved samples, and negative logFC represents lower abundance in starved replicates. “*” indicates adjusted *p*-value ≤ 0.05.

Of the 69 *M. sexta* neuropeptides in the assembled list, 20 were detected from prior midgut transcriptome data (Table 2). Of these, six were detected in the midgut proteome in the current work (Table 2, Figure 7). Neuropeptide F2 (NPF2, annotated as pro-neuropeptide Y (NPY)) was significantly more abundant (3.2-fold) in starved caterpillars in the 24 h only comparison. NPF1 was also more abundant in starved caterpillars in the combined analysis (1.2-fold), as was myosuppressin in the 24 h only (3.1-fold) and combined (2.1-fold) analyses compared with the fed samples. Insulin-associated insulin-like growth factor (IGF) 2 mRNA-binding protein 1 was more abundant in starved caterpillars (2.0-fold) in the 48 h only comparison. Further, an IGF1 receptor was significantly more abundant in the starved samples across all three analyses (combined: 2.5-fold; 24 h: 3.1-fold; 48 h: 2.0-fold). The neuropeptide orcokinin and the insulin-associated IGF-binding protein (IGFBP) complex acid labile subunit were also detected but were not significantly different between the starved and fed samples in any of the analyses (Figure 7).

## 4. Discussion

The insect midgut communicates the status of incoming nutrients to other body organs by releasing neuropeptide hormones, which helps maintain homeostasis during stressful conditions [3]. Therefore, a DIA proteomics approach was used as a broad method to understand the changes in the midgut under starvation stress and the impact on neuropeptide hormone signaling in *M. sexta*. Identifying changes in the midgut during nutritional stress can help elucidate mechanisms used in insects to cope with starvation. In the current work, 3047 proteins were identified across fed and starved fourth-instar *M. sexta* caterpillar samples. Generally, a greater number of proteins were detected in the fed samples compared to the starved samples.

### 4.1. Neuropeptide Hormones

Six neuropeptides or signaling cascade proteins were detected in the *M. sexta* midgut proteome dataset of the current study (Table 2; Figure 7). This is considerably less than the 20 neuropeptide hormone transcripts detected in *M. sexta* larval midgut transcriptomes [14]. A wide range of developmental conditions were sampled in prior transcriptomes, including non-feeding periods preceding and following larval molt, and expression may vary with physiological status. However, another larval midgut transcriptome examining *Spodoptera exigua* also detected several of the same and a few additional neuropeptide hormones including allatostatin (multiple forms: A, C1, C2, and CC), allatotropin, CCHamide (1 and 2), corticotropin-releasing-factor-like-diuretic hormone 44 (DH44), diuretic hormone 31/calcitonin-like peptide (DH31), diuretic hormone 45 (DH45), myosuppressin, NPF1, orcokinin (1 and 2), proctolin, short neuropeptide F (sNPF), and tachykinin [21]. While it is unclear whether the midgut actually translates detectable amounts of all of these peptides, particularly those detected at low transcript abundance, direct extraction, immunostaining, or *in situ* hybridization of several of these neuropeptide hormones in *M. sexta* or other lepidopterans [38,39,40] suggest that many are actively expressed in enteroendocrine cells or the innervation of the midgut. However, small peptides (such as sNPF) contain limited, if any, trypsin cleavage sites, may not have adhered to the column, or were below the mass range scanned in the current study. In addition, some peptides may be too low in abundance to be detected with DIA. DIA is an unbiased, reproducible, and sensitive method that, in addition to data-dependent acquisition (DDA), can detect low-abundance proteins and cover a higher dynamic range for quantitative proteomics [32,41,42,43]. The DIA approach used in this study permitted the broadest range of global detection for neuropeptides while also enabling quantitation, which provides a starting point for the examination of multiple midgut neuropeptide hormones simultaneously. A more targeted approach for specific peptides not detected in the current study may be required for future studies yet may sacrifice the broad-spectrum data collection of other midgut proteins.

Among the most abundant neuropeptide hormones detected in our proteome, NPF1 and NPF2 belong to the neuropeptide Y superfamily and are involved in feeding, food choice, and food-seeking behaviors, among other functions [44,45,46,47]. However, the role of midgut-derived NPF has been little studied, but recent work on *D. melanogaster* suggests that it has a role in reproduction [48]. In addition, midgut NPF has an incretin-like function, involved in the secretion of ILPs by the corpora cardiaca and the suppression of adipokinetic hormone (AKH) [10]. It also stimulates sugar satiation and a switch to protein-rich diets in newly mated *D. melanogaster* females [12]. In Lepidoptera, midgut NPF signaling has been linked to the production of α-amylase and lipase in the midgut [49]. In the current work, NPF1 was significantly more abundant in starved larvae in the combined analysis, and NPF2 was significantly more abundant in starved larvae in the fed 24 h vs. starved 24 h analysis. Previous work has reported a slight increase in NPF transcript abundance in the midgut under starvation in some insects [46,50], although this is not consistent for all insects [10,12]. However, a buildup of NPF in midgut endocrine cells under starvation conditions has been demonstrated by densitometry in *D. melanogaster* and suggests that NPF release, rather than synthesis, is regulated, allowing NPF to accrue in the enteroendocrine cells of starved midguts [10,12]. NPF may, therefore, be primed for a robust release from the midgut when an appropriate food stimulus becomes available to the starved insect. A similar dynamic of NPF1 and NPF2 accrual may have occurred in the current study. Further, a drastic drop in midgut NPF1 levels in the corn earworm, *Helicoverpa zea*, was detected at the onset of gut purge, as quantified by radioimmunoassay [45]. This also suggests that NPF synthesis and release from the midgut is tied to the nutritional status of midgut contents and development in Lepidoptera. The use of mass spectrometry in the current work highlights another potential tool for further studying the dynamics of NPF and other neuropeptide hormones in the midgut and other tissues in insects.

Myosuppressin (also known as FLRFamide) expression was previously described in *M. sexta* midgut endocrine cells using *in situ* hybridization [51], and this peptide inhibits feeding in other lepidopterans such as *Spodoptera littoralis* [52] and *Bombyx mori* [53]. In cockroaches, feeding inhibition by myosuppressin was suggested to be driven by the ability of myosuppressin to prevent peristaltic transport of food in the gut [54]. Several other digestive functions have been suggested for myosuppressin in insects, including enzyme secretion from the midgut [55,56] and inhibition of ion transport across the midgut epithelium [57]. Myosuppressin amounts increased in *M. sexta* midguts during starvation in the current study, which suggests thatpeptide is accruing rather than being released, similar to NPF1 [10,12]. Suppressing myosuppressin release in starved *M. sexta* may make sense if myosuppressin has the same feeding inhibition and digestive roles in *M. sexta* that are noted in other insects. However, myosuppressin is also expressed in the nervous system, and other functions, such as inhibiting the prothoracic gland during diapause, have been noted [58]. In contrast, a role for orcokinin in stimulating the prothoracic gland was demonstrated in *B. mori* [59]. Other diverse functions of orcokinin have been explored in disparate insects and include roles in ecdysis [60], reproduction [61,62], or predator-avoiding behaviors [63], among other functions. However, this neuropeptide overall remains understudied, and a receptor for orcokinin has not yet been identified [62]. Further, no work to date has disentangled the roles of midgut-derived orcokinins from those expressed in the nervous system. In the current study, levels of orcokinin in the midgut remained unchanged under starvation conditions, providing few clues as to its role besides that its release in *M. sexta* may not be tied directly to the presence or absence of food in the gut.

### 4.2. Impact of Starvation on Other Hormones

Three enzymes catalyze the degradation of JH in Lepidoptera: JH esterase (JHE), JH epoxide hydrolase (JHEH), and JH diol kinase (JHDK) [64]. JHEH and JHE play critical roles in reducing JH titers and instigating the release of PTTH and the subsequent rise in ecdysteroids initiating the molt to a pupa [65]. Although JH has been extensively examined in *M. sexta*, the focus has primarily been on its role in the transition from final instar larvae to the pupal state [64]. JHE levels in *M. sexta* are dependent on feeding states and reach maximum levels in fifth instars to eliminate JH when caterpillars reach critical weight before pupation [66]. Less explored are the roles of JH in younger instars, besides maintaining larval characteristics during molting. In this work, fourth-instar larvae were specifically chosen to mitigate the confounding influence of developmental changes incurred in the transition from larva to pupa. Interestingly, JHE was more abundant in the midguts of starved caterpillars in the current study, and JHEH was less abundant. An increase in JHDK was also noted in the midguts of starved *B. mori* caterpillars compared to fed ones [67]. Also, high JH titers were associated with decreased feeding in *Ostrinia furnicalis*, the Asian corn borer, and these levels were under the control of brain NPF [68]. Whether JH-degrading enzymes are synthesized to a greater or lesser degree or are merely stored in the midgut and are not released into the hemolymph was not determined in the current study but is an area for further exploration. Further, how exactly these different enzymes modulate JH titers is worthy of further study. JHEH, for instance, has different enzymatic activities on different forms of JH, which may have other influences beyond control of larval–pupal transition [64]. Staying in the same larval instar in nutritionally deficient states is a typical response in *M. sexta* caterpillars to nutrient stress and often supernumerary larval instars compensate for limited nutrition [69]. The current data hint at mechanisms that may modulate this response.

### 4.3. Metabolism and Digestive Enzymes

A primary function of the insect midgut is to break down and absorb ingested nutrients in the gut lumen, and many digestive enzymes have been identified in *M. sexta* [70,71,72]. Proteomics has been used to identify and localize digestive enzymes in other lepidopterans as well [73,74]. In addition, numerous other non-digestive catabolic proteins have been identified in *M. sexta,* likely for cellular homeostasis and other physiological functions [75]. These classes of enzymes were not differentiated in the current study, but as a whole, protein-, carbohydrate-, and lipid-degrading proteins were less abundant in starved midgut samples (Table 1). Mitochondrial proteins and pathways were also generally less abundant in starved caterpillars (Table 1; Figure 3, Figure 4 and Figure 5). This was not unexpected, as when an insect experiences starvation stress, digestive enzymes are not immediately needed, and energy use and growth are curtailed [76,77,78,79]. Instead, stored reserves are accessed to maintain homeostasis [80] as reflected in the identified KEGG pathways upregulated for oxidative phosphorylation and amino acid degradation in the starved caterpillars (Figure 4, Figure 5 and Figure 6). In particular, fructose and mannose metabolism pathways were more abundant in the starved samples in the combined and 48 h analyses, suggesting that these sugars are used as an energy substrate in starved caterpillars in this stage of starvation (Figure 4, Figure 5 and Figure 6). Although not the primary focus of the current study, this description provides baseline information on digestive enzymes and energy homeostasis that may lead to further explorations of digestion–energy dynamics in starving midgut tissue in *M. sexta*.

### 4.4. Feeding Variation

Starved replicates clustered together in the PCA analysis of the current study, irrespective of time, and may indicate that the length of starvation does not influence the midgut response to starvation or that 48 h was not enough time to induce a more severe response change (Figure S1). Previous work suggested differences in mobilization of energy reserves in the first 1–2 days of starvation versus longer starvation periods [16]. Conversely, PCA analysis demonstrated an overlap in feeding samples between 24 h and 48 h (Figure S1). There could be several reasons for this. For instance, feeding in *M. sexta* is not continuous and occurs in bouts followed by periods of apparent inactivity [81], although the caterpillar is likely engaged in the internal processes of digestion during so-called inactivity [82,83]. Even small breaks in feeding (15–45 min) can induce the activation of enzymes such as glycogen phosphorylase in the fat body [84]. Therefore, proteome composition may be influenced by whether the insect is actively ingesting fresh food, digesting already consumed food, or defecating; its meal size; or whether it is searching for food for the next round of eating, among other factors. Carefully controlling the harvesting of caterpillars for proteomics based on the precise timing of feeding is an opportunity for future exploration of the complex process of food consumption, digestion, and excretion.

## 5. Conclusions

The quantitative proteome generated in this study provides an overview of the dynamics of the midgut in fed and starved *M. sexta* larvae. An extensive diversity of proteins associated with the starvation response was identified, and six neuropeptide hormones or associated signaling cascade molecules with a putative connection to starvation stress were also recognized. NPF1 was significantly more abundant in starved larvae in the combined fed vs. starved analysis, suggesting a role in starvation physiology in *M. sexta*. Future studies confirming starvation-associated NPF1 level changes in the hemolymph may provide clues for the role of midgut-released NPF1 and other neuropeptide hormones in *M. sexta* caterpillars.

## Figures and Tables

**Table 1:** Selected significantly different protein abundance (adjusted *p*-value ≤ 0.05) across 24 h, 48 h, and combined comparisons, categorized by functional group subsections. Positive log fold changes (logFC) represent higher abundance in starved replicates, and negative fold changes represent lower abundance in starved replicates. Remaining significant proteins are listed in Table S3.

Protein ID	Protein Name	24 h logFC	48 h logFC	Combined logFC
**Protein degradation**				
XP_030030816.2	Mitochondrial-processing peptidase subunit beta	1.5	2.1	1.8
XP_030031119.2	Probable aminopeptidase NPEPL1	1.3	1.1	1.2
XP_030028976.2	Zinc carboxypeptidase	−1.4	−2.2	−1.8
XP_030023348.2	Carboxypeptidase B	−1.9	−2.9	−2.4
XP_030027851.2	CAAX prenyl protease 1 homolog	−2.1	−2.1	−2.1
XP_037300887.1	Antichymotrypsin-2-like	−2.4	−4.0	−3.2
XP_030024172.2	Lysozyme	−2.3	−4.8	−3.6
XP_030021372.2	Trypsin, alkaline C-like	−3.7	−6.1	−4.9
**Sugar degradation**				
XP_030027415.1	Maltase A1	−2.0	−4.2	−3.1
XP_037295803.1	Lactase–phlorizin hydrolase	−2.7	−5.7	−4.2
XP_037295801.1	Lactase–phlorizin hydrolase	−4.2	−7.2	−5.7
**Lipid degradation**				
XP_030034911.2	Venom carboxylesterase 6	1.7	2.2	1.9
XP_030029542.2	Pancreatic lipase-related protein 2-like	−4.3	−4.6	−4.4
**Mitochondrial**				
XP_037294842.1	Alanine–glyoxylate aminotransferase 2	2.6	3.1	2.8
XP_030027392.1	Cytochrome c oxidase subunit 4 isoform 1	1.9	2.3	2.1
XP_030029341.1	39S ribosomal protein L41	−1.1	−1.2	−1.1
XP_030024656.1	39S ribosomal protein L37	−1.4	−2.0	−1.7
XP_030035113.2	3-ketoacyl-COA thiolase	−1.5	−2.6	−2.1
XP_030031455.1	Mitochondrial glutamate carrier 1	−2.1	−3.0	−2.6
XP_030026609.1	Short-chain specific acyl-COA dehydrogenase	−3.0	−2.7	−2.9
XP_037294570.1	39S ribosomal protein s18a	−3.7	−3.4	−3.6
XP_037295058.1	3-ketoacyl-coa thiolase	−4.0	−3.7	−3.8
**Metabolism**				
XP_030029916.1	Diacylglycerol O-acyltransferase 1	−1.4	−1.6	−1.5
XP_037292967.1	Probable 3-hydroxyacyl-coa dehydrogenase B0272.3	−1.3	−1.9	−1.6
XP_030023792.1	Hydroxysteroid dehydrogenase-like protein 2	−2.1	−1.6	−1.8
XP_030023969.1	3-hydroxyacyl-coa dehydrogenase type-2	−1.8	−1.9	−1.9
XP_030032134.1	Epidermal retinol dehydrogenase 2	−2.3	−1.7	−2.0
XP_030028836.1	D-altritol 5-dehydrogenase	−2.5	−2.1	−2.3
XP_030021537.1	1-acyl-sn-glycerol-3-phosphate acyltransferase gamma	−2.6	−2.0	−2.3
XP_030026278.2	Aldehyde dehydrogenase, dimeric NADP-preferring	−2.5	−2.6	−2.6
XP_030037221.2	Estradiol 17-beta-dehydrogenase 8	−2.9	−3.4	−3.2
XP_030038272.1	15-hydroxyprostaglandin dehydrogenase [NAD (+)]	−2.5	−4.2	−3.4
**Juvenile hormone**				
XP_030040069.2	Juvenile hormone esterase	1.9	4.4	3.1
XP_037295223.1	Juvenile hormone esterase-like	1.9	2.8	2.4
XP_030032265.2	Juvenile hormone epoxide hydrolase	−1.7	−2.5	−2.1
**Cytochromes**				
XP_030034471.1	Cytochrome b5	1.9	2.4	2.1
XP_030040484.1	Cytochrome c-like	−1.1	−1.4	−1.3
XP_030025415.2	NADH-cytochrome b5 reductase 2	−1.4	−1.3	−1.3
**Basement membrane assembly (laminin)**				
XP_030028135.1	Laminin subunit beta-1	2.3	1.7	2.0
XP_037299829.1	Laminin subunit gamma-1	2.4	1.7	2.0
XP_037299653.1	Laminin subunit alpha-like	2.1	1.7	1.9

**Table 2:** Peptide hormones detected in *M. sexta* larval midguts via proteomics (current study) or datamined from previous midgut transcriptomes [14].

Peptide	*M. sexta* Proteome	*M. sexta* Transcriptomes
Allatostatin A (helicostatins)		++
Allatostatin C		+++
Bombyxin-related peptide A	na	+++
Bombyxin-related peptide A	na	+
CCHamide-1		+
CCHamide-2		++
Corticotropin-releasing factor-like/diuretic hormone 44		+
Diuretic hormone class 2/diuretic hormone 31/Calcitonin-like		+
Ecdysis-triggering hormone		+
IDLSRF-like peptide		+
Insulin-like growth-factor-binding protein complex acid labile subunit	+	+++
Insulin-like growth factor 2 mRNA-binding protein 1	+	+++
Myosuppressin	+	+++
Neuropeptide-like 4		++++
NPF1 (a and b)	+	+++
NPF2	+	++
Orcokinin	+	++++
RYamide neuropeptides		+
sNPF		+
Tachykinin		+++

For the transcriptome, ++++ = >1000; +++ = >100; ++ = >50; + = >10 FPKM in midgut summed across all larval stages. “na“ indicates the peptide is not annotated in the JHU_Msex1.0 assembly. See Table S1 for a full list of annotated *M. sexta* neuropeptides.

## Data Availability

The data that supports the findings of this study are available in the manuscript and/or are available upon request from the authors.

## Supplementary Materials

The following supporting information can be downloaded at https://www.mdpi.com/article/10.3390/insects15050325/s1: Figure S1: Principal component analysis of protein intensities representing distinct grouping of fed vs. starved samples. Fed 24 h (red) and 48 h (green) grouped separately from starved 24 h (blue) and 48 h (purple) samples. Table S1: List of neuropeptides in *M. sexta* as annotated in the genome assembly JHU_Msex_v1.0 [23] (Gershman et al., 2021). Table S2: Diet ingredients and preparation for the *M. sexta* colony rearing in this study. Table S3: Complete list of significantly different *M. sexta* midgut proteins detected by DIA spectroscopy in this study. Proteins are sorted by the number of analyses where a significant change in abundance was detected (adjusted *p*-value ≤ 0.05) and then by log fold changes (compared to starved samples; i.e., positive values indicate proteins more abundant in starved samples versus fed). Significantly different proteins are highlighted: blue for 24 h analysis; yellow for 48 h analysis; green for combined analysis.
