# Supplementary material for: Starvation-Induced Changes to the Midgut Proteome and Neuropeptides in Manduca sexta
Source: Insects. 2024 May 2;15(5):325. doi: 10.3390/insects15050325 (PMC11121805; doi:10.3390/insects15050325)
Supplement: Supplementary file 1 [file insects-15-00325-s001.zip › Table S1. Manduca neuropeptides 03-22-24 .pdf]

Table S1: List of neuropeptides in *M. sexta* as annotated in the genome assembly JHU_Msex_v1.0 (Gershman et al., 2021).

Peptide	Gene ID	Protein ID
Adipokinin hormone (AKH)	LOC115443027	XP_030024155.2, XP_037297759.1
Allatostatin-A	LOC115449048	XP_030032599.2
Allatostatin-C	LOC115456115	XP_030040870.1
Allatostatin-CC	LOC115456123	XP_037294552.1, XP_037294552.1
Allatotropin-like (AT)	LOC115441199	XP_037294817.1
Bombyxin-related peptide A	LOC115447814	XP_030030912.2
Bombyxin-related peptide A	LOC115447816	XP_030030914.2
Bombyxin-related peptide A	LOC115447817	XP_037294793.1
Bombyxin-related peptide A	LOC115448889	XP_037297313.1, XP_037297315.1, XP_037297316.1, XP_037297314.1, XP_037297311.1
Bombyxin-related peptide A-like	LOC115447818	XP_030030916.2
Bombyxin-related peptide A-like	LOC115447819	XP_030030917.2
Bombyxin-related peptide A-like	LOC115447821	XP_030030918.2
Bombyxin-related peptide B-like	LOC115447807	XP_030030906.2
Bombyxin-related peptide B-like	LOC115447808	XP_030030907.2
Bombyxin-related peptide B-like	LOC115447809	XP_030030908.2
Bombyxin-related peptide B-like	LOC115447810	XP_030030909.2
Bombyxin-related peptide B-like	LOC115447811	XP_030030910.2
Bombyxin-related peptide B-like	LOC115447813	XP_030030911.2
Bombyxin-related peptide B-like	LOC115447823	XP_030030920.2
Bombyxin-related peptide B-like	LOC119189397	XP_037294792.1
Bursicon-like	LOC115445641	XP_030027854.2
CAPA peptides	LOC115444678	XP_030026443.1
Cardioactive peptide-like (CCAP)	LOC115450766	XP_030034735.1
CHH-like protein / Ion transport peptide	LOC115451886	XP_037295133.1, XP_037295131.1, XP_037295130.1
Corticotropin-releasing factor-binding protein-like / diuretic hormone 44	LOC119188406	XP_037296706.1
Diuretic hormone 1-like	LOC115450429	XP_030034305.1, XP_030034306.1, XP_037300155.1
Diuretic hormone 45	LOC115450420	XP_030034294.1
Diuretic hormone class 2 / Diuretic hormone 31/ Calcitonin-like	LOC115440347	XP_030020468.2
Diuretic hormone class 2-like	LOC119192129	XP_037301862.1
Ecdysis-triggering hormone (ETH)	LOC115441646	XP_030022367.1
Eclosion hormone-like (EH)	LOC115448567	XP_030031898.2, XP_037296919.1
FMRFamide-related peptides	LOC115445022	XP_030026977.1
IDLSRF-like peptide	LOC115440965	XP_030021367.1
Insulin-like growth factor 2 mRNA-binding protein 1	LOC115441152	XP_030021654.1, XP_030021655.1, XP_030021656.1, XP_030021657.1, XP_030021658.1, XP_037299926.1
Insulin-like growth factor 2 mRNA-binding protein 2	LOC119191761	XP_037301523.1
Insulin-like growth factor-binding protein complex acid labile subunit	LOC115442831	XP_030023865.1

Insulin-related peptide 2	LOC115456431	XP_030041353.1, XP_030041354.1, XP_037296580.1
ITG-like peptide	LOC115439790	XP_030019642.1, XP_030019643.1
ITG-like peptide	LOC119189620	XP_037295713.1, XP_037295714.1
Leucokinin	LOC115440945	XP_030021331.2
Myosuppressin (MS)	LOC115440942	XP_030021327.1, XP_030021328.1
Natalisin (NTL)	LOC115455961	XP_030040656.1, XP_030040654.1, XP_030040655.1
Neuropeptide CCHamide-1	LOC115447790	XP_030030881.2, XP_030030889.2
Neuropeptide CCHamide-2	LOC115447598	XP_030030599.1
Neuropeptide F 1 (a and b)	LOC115440163	XP_030020215.2, XP_037297084.1
Neuropeptide IMFamide	LOC115449258	XP_030032889.2, XP_030032890.2, XP_030032892.2, XP_037296121.1
Neuropeptide SIFamide	LOC115449261	XP_037296122.1
Neuropeptide-like 4	LOC115450709	XP_030034651.1
Neuropeptide-like 4	LOC115450699	XP_030034640.1
Neuropeptide-like 4	LOC119191143	XP_037300931.1
Neuropeptide-like precursor 1 (NPLP1)	LOC115455839	XP_030040440.2
Orcokinin	LOC115440296	XP_030020405.2
Partner of bursicon / Bursicon beta subunit	LOC115445642	XP_030027856.2
PBAN-type neuropeptides	LOC115453743	XP_030038326.2
Pro-corazonin	LOC115443369	XP_037296059.1
Pro-neuropeptide Y (NPF2/NPY)	LOC115448624	XP_030031971.1
Proctolin	LOC115442035	XP_030022852.2
Prothoracicostatic peptide (Allostatin B)	LOC115442476	XP_030023380.2, XP_037292238.1, XP_037292237.1
Prothoracicotropic hormone (PTTH)	LOC115442546	XP_030023461.1, XP_030023462.1
Putative neuropeptide precursor protein	LOC115440253	XP_030020340.2
Ryamide neuropeptides	LOC115439774	XP_030019620.1
Short neuropeptide F (sNPF)	LOC115445847	XP_030028178.1, XP_030028186.1
Tachykinins	LOC115455967	XP_030040667.1
Thyrostimulin alpha-2 subunit / glycoprotein hormone alpha 2	LOC115453281	XP_030037834.2
thyrostimulin beta-5 subunit	LOC115445425	XP_030027548.1
U-scoloptoxin(20)-Cw1a / Trissin	LOC115450769	XP_030034738.1
U-scoloptoxin(20)-Sm1a / Trissin	LOC115450754	XP_030034722.1
Trissin	LOC115450767	XP_030034736.1
Trissin	LOC115450768	XP_030034737.1
