# Supplementary material for: Starvation-Induced Changes to the Midgut Proteome and Neuropeptides in Manduca sexta
Source: Insects. 2024 May 2;15(5):325. doi: 10.3390/insects15050325 (PMC11121805; doi:10.3390/insects15050325)
Supplement: Supplementary file 1 [file insects-15-00325-s001.zip › Table S2. Manduca diet.pdf]

Table S2: Diet ingredients and preparation for *Manduca sexta* colony rearing in this study.

Group 1	
Casein	120 g
Toasted Wheat germ	340 g
Torula yeast	40 g
Cholesterol	12 g
Sucrose	120 g
Wesson salt	44 g
Linseed oil	20 ml
Alphacel	20 g
Distilled Water	1400 ml
**Group 2**	
Distilled Water	1600 ml
Agar	65 g
**Group 3**	
Ascorbic Acid	20 g
Aureomycin (Chlortetracycline)	1.2 g
Methyl paraben	6 g
Choline chloride	4 g
Sodium propionate	6.2 g
Sorbic acid [(2,4)-hexadienoic acid]	8 g
Vanderzants' Vitamins	52.5 g
**Other components**	
Distilled water (cold)	440 ml
37% Formaldehyde	2 ml
4M KOH	20 ml

Diet preparation:

Group 1 ingredients were added to a blender and blended at LOW speed for 5 min. Group 2 (agar) was prepared in a separate container and was completely dissolved in 1,600 ml of boiling water, stirring constantly. This was allowed to cool to ~60 °C, then was added to the blender and mixed at medium speed. Group 3 ingredients were mixed in a separate container. Once Group 2 ingredients were cooled to ~45 °C, Group 3 ingredients were added with 440 ml water and blended. KOH and formaldehyde were then added and blended for ~2 min until smooth in consistency. Diet was immediately poured into pans (1-3 cm depth) and stored at 4˚C until use. Diet was kept for up to 1 week before discarding.
