# Supplementary figures and images for: Starvation-Induced Changes to the Midgut Proteome and Neuropeptides in Manduca sexta
Source: Insects. 2024 May 2;15(5):325. doi: 10.3390/insects15050325 (PMC11121805; doi:10.3390/insects15050325)

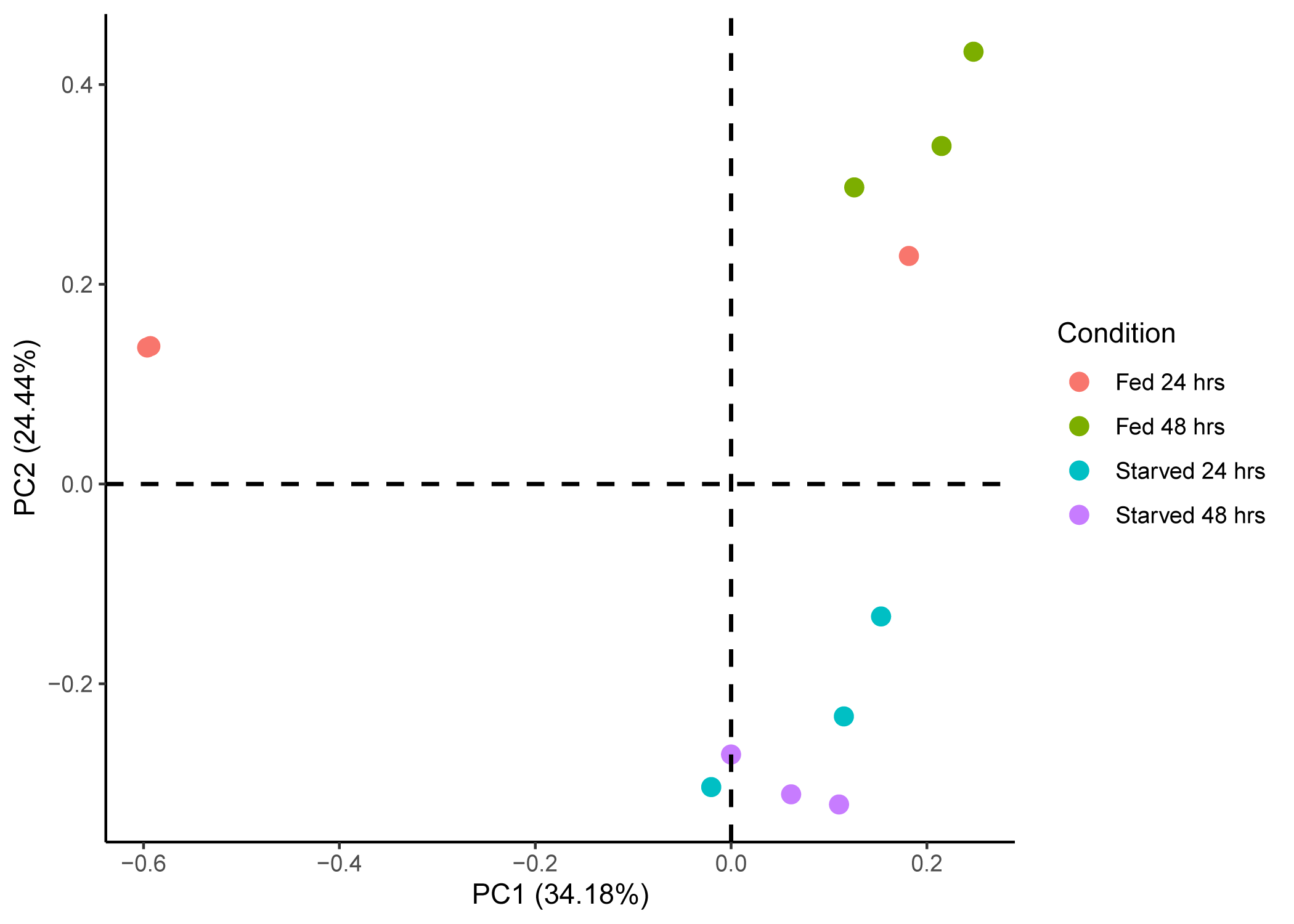

Supplement: Supplementary file 1 [file insects-15-00325-s001.zip › Figure S1. PCA plot.tif]
